# Sensitivity to the sonority sequencing principle in rats (*Rattus norvegicus*)

**DOI:** 10.1038/s41598-023-44081-y

**Published:** 2023-10-09

**Authors:** Chiara Santolin, Paola Crespo-Bojorque, Nuria Sebastian-Galles, Juan Manuel Toro

**Affiliations:** 1 Center for Brain and Cognition, Universitat Pompeu Fabra, Barcelona, Spain; 2 Catalan Institution for Research and Advanced Studies, Barcelona, Spain

**Keywords:** Evolution, Psychology

## Abstract

Albeit diverse, human languages exhibit universal structures. A salient example is the syllable, an important structure of language acquisition. The structure of syllables is determined by the Sonority Sequencing Principle (SSP), a linguistic constraint according to which phoneme intensity must increase at onset, reaching a peak at nucleus (vowel), and decline at offset. Such structure generates an intensity pattern with an arch shape. In humans, sensitivity to restrictions imposed by the SSP on syllables appears at birth, raising questions about its emergence. We investigated the biological mechanisms at the foundations of the SSP, testing a nonhuman, non-vocal-learner species with the same language materials used with humans. Rats discriminated well-structured syllables (e.g., *pras*) from ill-structured ones (e.g., *lbug*) after being familiarized with syllabic structures conforming to the SSP. In contrast, we did not observe evidence that rats familiarized with syllables that violate such constraint discriminated at test. This research provides the first evidence of sensitivity to the SSP in a nonhuman species, which likely stems from evolutionary-ancient cross-species biological predispositions for natural acoustic patterns. Humans’ early sensitivity to the SSP possibly emerges from general auditory processing that favors sounds depicting an arch-shaped envelope, common amongst animal vocalizations. Ancient sensory mechanisms, responsible for processing vocalizations in the wild, would constitute an entry-gate for human language acquisition.

## Introduction

Despite the important variability observed across human languages, some common structures can be found amongst them. Languages are, indeed, rooted in universal constraints that shape their structures and learnability. A fundamental, unsolved question is how such universal linguistic constraints emerge in humans, and what biological mechanisms might be at their basis. Here we investigate whether a well-documented constraint that operates across languages, the Sonority Sequencing Principle (SSP), arises from evolutionary-ancient sensory processing mechanisms. Evidence of sensitivity to the SSP can be observed from birth, as human neonates process sonority differences defining syllables. We explore whether such early sensitivity to the SSP emerges from general processing of the acoustic input, testing a nonhuman species.

**Figure 1:**
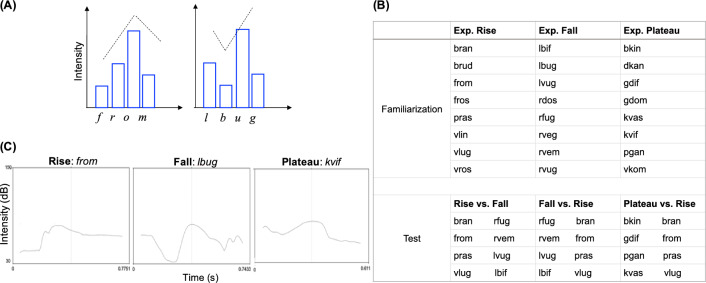
Changing sonority across syllables. (**A**) Schematic representation of the SSP. Left: *from*, example of a well-structured syllable matching the SSP. The intensity of /f/ and /r/ rises until /o/ is reached, and subsequently falls. Sonority degrees (s) of such phonemes are: /**f**/ s = 1, /**r**/ s = 3, /**o**/ s = 5, /**m**/ s = 2. The highest the value, the most sonorant the phoneme^14^; vowels are the most sonorant of all. Right: *lbug*, example of an ill-structure syllable violating the SSP. Sonority degrees (s) are: /**l**/ s = 3, /**b**/ s = 1, /**u**/ s = 5, /**g**/ s = 1. The drop of intensity is caused by the second phoneme /b/ having a lower intensity than the initial phoneme /l/. (**B**) Syllables used in the three experiments, during Familiarization (above) and Test (below). Syllables are retrieved from^13^. (**C**) Examples of intensity contours of syllables used in the three experiments (one per category: Rise, Fall, Plateau). Contours are plotted in Praat (version 6.1.53). Y axis indicates intensity (dB), X axis indicates time, the vertical dotted line indicates the mid of the temporal window. Note that Plateau syllables are not completely flat, but show a flatter intensity contour at the onset (before the mid line) with respect to Rise and Fall syllables.

The SSP is a phonological constraint that determines the internal structure of syllables, shared across most languages of the world^1,2^. From birth, young infants spontaneously process the speech stream as units that roughly correspond to syllables^3,4^, which become important elements of subsequent linguistic processing^5,6^. Newborns automatically synchronize their brain activity to the syllable frequency rate^7^ in a similar way as it is observed in adults^8^. The SSP imposes restrictions on how phonemes (consonants and vowels) are combined into syllables based on their intensity, which tends to increase towards the nucleus (where the vowel is) and decline after it^9–11^. Well-structured syllables thus allocate the most sonorous phoneme (the vowel) at their nucleus, and the least sonorous ones (typically consonants) at their edges, generating an intensity pattern with an arch shape (Fig. 1A).

Linguistic research shows that well-structured syllables are very frequent across languages with respect to syllables that do not conform to the SSP (although languages violating SSP are not rare; e.g.^12^). Two-to-five day-old neonates are sensitive to violations of the SSP^13^. In this study, syllables were classified based on the different degree to which they conformed to the SSP. *Rise* syllables depicted a prototypical rise of intensity from onset to nucleus, a frequent pattern across languages (e.g., *pras*). *Plateau* syllables showed a flat intensity from onset to nucleus (e.g., *gdif*). This pattern complies with the SSP, as the intensity does not decrease before the vowel. For instance, the English word *stem* has an initial consonant cluster /*st*/ with flat intensity. *Fall* syllables, instead, presented a reversed (falling) pattern of intensity breaking the SSP (e.g., *lbug*). Results show distinct neural responses to well-structured vs. ill-structured syllables, suggesting that precursors of the SSP are in place at the very onset of language development. Human adults perceive violations of the SSP even in languages they do not speak. Korean speakers are sensitive to the well-formedness of English consonant clusters (phonologically “repairing” the ill-structure *lbif* as *lebif*) even though their native language does not allow consonant clusters^14,15^; for examples in other languages^16–18^. The existing evidence points to universal processing of the SSP, which is perceived from the early stages of human language acquisition.

The SSP generates an intensity pattern with a characteristic arch-shaped envelope that mimics the melody of several animal vocalizations (e.g., among others, bird songs, nonhuman primate alarm calls, cat meows^19,20^). For instance, budgerigars, a parrot species, organize their own songs into short units that share sound pattern biases (e.g., start with plosive-like sounds, followed by vowel-like sounds) with human syllables^21^. We propose that humans’ sensitivity to the SSP at birth, and in languages they do not have experience with, emerges from general auditory processing mechanisms favoring sounds depicting an arch-shaped envelope. Sounds that break such envelope (e.g., *rban*, *lbif*, ill-structured syllables violating the SSP) are likely more difficult to process and parse into units. Such general processing could constitute the biological basis of the SSP in human languages. If this is the case, it would not be surprising to find an advantage for syllables with a rising intensity contour over syllables with a falling intensity contour in other animal species. Research on other cognitive domains shows that nonhuman species are sensitive to general perceptual constraints operating on the sensory input. Notable examples include the Gestalt principles^22^ which allow animals to process visual signals as whole units^23–25^ or decompose a jumble of sounds into auditory objects^26,27^. Gestalt principles provide animals with powerful mechanisms to quickly organize visual and auditory signals, grouping elements together and separating others, on the basis of physical cues provided by the inputs (e.g., acoustic frequency separation, common contours of visual items^28^).

The aim of the present research is to investigate the biological mechanisms at the roots of the SSP, testing Long-Evans rats (*Rattus norvegicus*) in the ability to discriminate human syllables that match *versus* violate the SSP. Importantly, to maximize the possibility of drawing parallels across species, we used the same language materials used with human neonates^13^: three types of syllables classified based on the different degree to which they conformed to the SSP (*Rise*, *Plateau*, *Fall*). Contrary to humans, rats are non-vocal-learners, being unable to imitate or learn to produce new sounds, which is instead a critical component of language and other forms of animal vocalizations^29,30^. Rats produce only two types of ultrasonic calls^31,32^. The 22 kHz calls are flat alarm signals with low peak frequency and duration up to 3 s. The 50 kHz calls are high-peaked affiliative calls with shorter duration (up to 150 ms), that can be flat or having some frequency oscillations. Both categories of calls are ultrasonic whistles produced by laryngeal muscles^33,34^ and do not require significant movements of the articulators (e.g., mouth, tongue). Previous research has shown that rats can compute different aspects of the speech, such as processing rhythmic cues that differentiate languages^35^, discriminating speech sounds^36^, detecting statistical co-occurrence of syllables forming words in continuous speech^37^, and processing prosodic contours^38^. Rats thus represent an excellent animal model for the proposed research. We conducted three experiments, each one with a separate group of rats. The animals were familiarized with CCVC (consonant–consonant–vowel–consonant) syllables. As in^13^, *Rise* and *Plateau* syllables complied with the SSP, *Fall* syllables did not (Fig. 1B,C). After familiarization, rats were tested with familiar and novel syllables. Novel syllables were formed by new combinations of the same phonemes composing familiarization syllables. Importantly, novel syllables had intensity patterns that rats did not experience during familiarization (see “Methods” for details).

## Results

**Figure 2:**
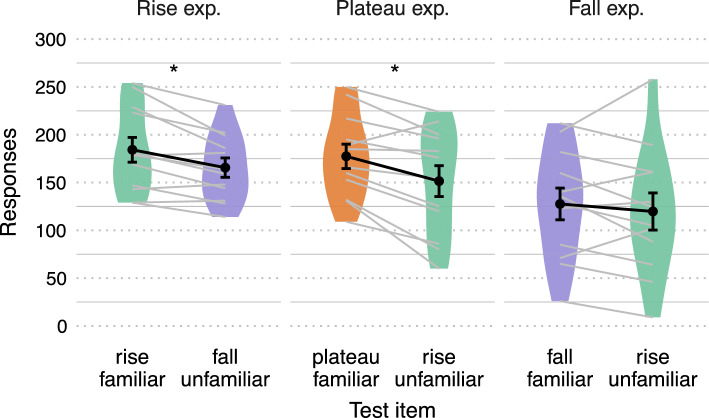
Test results. Number of nose-poking responses to syllables at test for Rise, Plateau and Fall experiments. Dots indicate mean responses, error bars indicate standard error of the mean. Grey lines connect individual responses to syllables presented at test in each experiment.

We measured discrimination between familiar and novel syllables at test as a function of the number of nose-poking responses for each syllable type, with paired-samples t-tests. We expected rats to produce a greater number of nose-pokes in response to the syllable type presented at familiarization, suggesting a preference for known stimuli. Results show that, in Rise and Plateau experiments, rats successfully discriminated between familiar and novel test syllables. In the Rise experiment, rats showed a significantly greater number of responses for *Rise* vs. *Fall* syllables (t(11) = 3.793**,** p = 0.003, *d* = 1.095). In the Plateau experiment, rats showed a significantly greater number of responses for *Plateau* vs. *Rise* syllables (t(11) = 3.681, p = 0.004, *d* = 1.063). Note that *Rise* syllables were familiar items for rats in the Rise experiment but novel items for rats in the Plateau experiment (see Fig. 1,B). Conversely, in the Fall experiment, rats did not show significant differences in responses to *Fall* vs. *Rise* syllables (t(11) = 0.903, p = 0.386, *d* = 0.261; see Fig. 2 and Table 1). This pattern of results demonstrates that rats recognized the intensity patterns to which they were familiarized, discriminating familiar vs. novel test syllables only when such patterns adhere to the SSP (i.e., *Rise* and *Plateau* syllables). We did not find any evidence suggesting that rats of the Fall experiment (familiarized to an intensity pattern that altered the envelope of properly-structured syllables) discriminated at test.

**Table 1:** Summary of mean responses by experiment and by test item.

	Experiment		
	Rise (rise vs. fall)	Plateau (plateau vs. rise)	Fall (fall vs. rise)
Familiarization	180.68 (65.56)	146.27 (73.54)	123.5 (66.78)
Test			
Familiar items	184.25 (44.92)	177.42 (44.14)	127.59 (57.46)
Novel items	165.58 (34.99)	151.5 (56.05)	119.75 (67.28)
Overall means	174.92 (40.52)	164.46 (51.09)	123.67 (61.32)

Standard deviations of the mean are indicated between parentheses.

**Figure 3:**
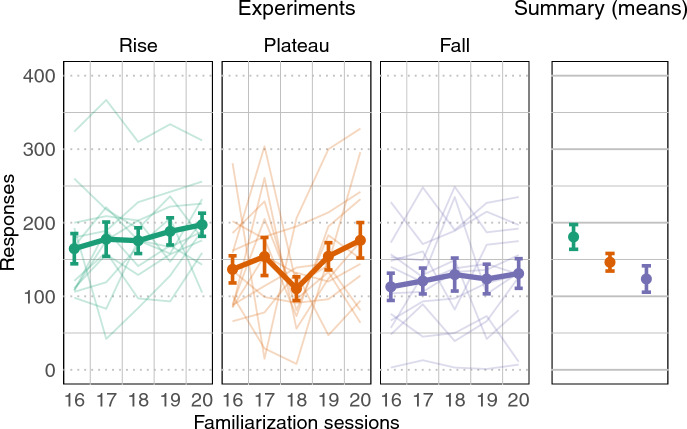
Familiarization results. *Experiments*: nose-poking responses during the last 5 sessions of familiarization of Rise (green), Plateau (orange) and Fall (purple) experiments. Dots indicate mean responses during a session, error bars indicate standard error of the mean. Faded lines indicate rats’ individual performance. *Summary*: mean number of responses across sessions for each experiment.

We also analyzed a potential processing advantage for the syllable type depicting the most natural intensity pattern (i.e., *Rise*), expecting an increased number of nose-pokes for such syllables during familiarization and test. The rationale of such analysis is based on previous research on nonhuman species and human infants revealing general preferences for natural patterns such as biological motion^39,40^ and face-like configurations^41,42^. We compared rats’ responses during the last five sessions of familiarization across experiments, with a one-way ANOVA. Results show a significant difference (F(2,33) = 3.317, p = 0.048, η^2^ = 0.17); pair-wise t-test comparisons show that rats familiarized with *Rise* syllables responded significantly more during familiarization than rats familiarized with *Plateau* (p = 0.01) and *Fall* syllables (p < 0.01). Rats familiarized with *Plateau* syllables also produced a greater number of nose-pokes with respect to rats familiarized with *Fall* stimuli although the difference does not reach statistical significance (p = 0.071; see Fig. 3 and Table 1). To clarify the role of potential pre-familiarization experiences that rats may have had (although note that rats were housed and tested in acoustically-controlled lab setting), we analyzed the first five sessions of familiarization across experiments with the same one-way ANOVA. As expected, results show no differences between the groups of rats at the beginning of familiarization (F(2,33) = 1.64, p = 0.209, η^2^ = 0.09) indicating no a priori bias for any of the syllable types. All the differences observed during the experiment can thus be attributed to the familiarization. A parallel analysis of the total number of nose-poking responses produced at test show significant differences across the three experiments (F(2,69) = 6.5905, p = 0.002, η^2^ = 0.16). Pair-wise t-test comparisons reveal that rats familiarized with *Fall* syllables responded significantly less than rats familiarized with *Rise* (p = 0.003) and *Plateau* (p = 0.012) syllables. In contrast, rats familiarized with *Rise* and *Plateau* syllables did not differ in their number of overall test responses (p = 0.486).

## General discussion

Our study demonstrates that rats can discriminate between syllables when they are familiarized with intensity contours that obey the SSP (instantiated by *Rise* and *Plateau* syllables). It also shows a sensitivity to syllables’ well-formedness as determined by sonority constraints, reflected in the spontaneous greater number of nose-pokes during familiarization. It is unlikely that rats at test were just responding to the familiarity of the stimuli. If this was the case, rats of the Fall experiment would have shown a greater number of responses for the test syllables to which they were familiarized (*Fall*), as occurred in the other experiments.

These findings fit well with the processing advantage for well-structured syllables observed in human neonates^13^ and adults regardless of their native languages^14,15^. Additionally, such findings point to the fact that the SSP not only facilitates the acquisition of syllabic structures in humans, but also favors the processing of certain acoustic patterns in other animals, specifically, those mirroring natural arch-shaped melodies of the wild. Melodic contours with an initial rise of intensity and a subsequent decline are often found in animal vocalizations, likely because of biomechanical constraints of sound production. At the onset of a vocalization produced through exhalation, such as human utterances or birdsong, the subglottal pressure in the vocal tract (behind the vocal folds) rises quickly and then drops. This causes an initial burst of intensity that generates the arch-shaped envelope^19–21^. Despite the presence of certain “ingressive” vocalizations, it is physiologically more efficient for humans and most mammals to vocalize while exhaling^43,44^. Rats only produce two types of ultrasonic whistles that do not significantly engage articulatory muscles. Therefore, it is plausible that rats relied on acoustic cues rather than articulatory information, to differentiate syllable types and better process well-structured ones. We interpret the fact that rats familiarized with *Fall* syllables did not show evidence of discrimination at test, and responded significantly less during familiarization, as indicating a potential difficulty in processing speech structures with multiple sonority peaks (as occur in syllables violating the SSP) that are less common in natural languages. More broadly, intensity patterns violating sonority constraints (hence, the arch-shaped envelope) are also unusual in animal vocalizations. Our findings align well with results on human language acquisition. Adults and 7-month-old infants process more easily words that start rather than end with higher intensity, as defined by the Iambic-Trochaic Law^45^. Similarly, prosodic contours characterized by a rise of intensity and longer duration at the end of a speech string promote learning of new words in 6-month-old infants^46^, toddlers^47^, and adults^48,49^.

The present research provides the first evidence of sensitivity to the linguistic Sonority Sequencing Principle in a nonhuman species. Such sensitivity would reflect general preferences for natural patterns also found in other domains. For instance, nonhuman species as well as human neonates spontaneously prefer biological motion, instantiated as point-light animation sequences generating the perception of a moving body^50^. Biological motion is preferred to backward motion or other unnatural types of movements^39,40,51,52^. Such preferences have been interpreted as emerging from predispositions for detecting patterns of movements predominantly found in nature. A similar bias is documented for face-like stimuli matching the prototypical (natural) configuration of facial features (e.g., upright vs. inverted faces^53–55^). We interpret our results within a similar framework: rats processed more easily well-structured syllables (conforming to the SSP) because the intensity envelope of such syllables prevails amongst the sounds of the environment.

Our findings demonstrate that sensitivity to some linguistic constraints, such as the SSP, stems from evolutionary-ancient biological predispositions shared with other species. In humans, such predispositions would allow the parsing of speech into fundamental linguistic units. Ancient sensory mechanisms, responsible for processing the calls of the wild, would thus constitute an entry-gate for human language acquisition.

## Methods

### Ethical statement

All the experiments and procedures reported in this paper were approved by the ethical committee of the Universitat Pompeu Fabra and the Generalitat de Catalunya, in compliance with Catalan, Spanish and European guidelines and regulations for the treatment of experimental animals. The protocol number assigned to these experiments is 10557. All methods were performed in accordance with relevant guidelines and regulations, and the study was conducted in compliance with the ARRIVE guidelines.

### Subjects

Thirty-six female Long-Evans rats (*Rattus norvegicus*) of 3 months of age were used in the study. Twelve rats were randomly assigned to each of the 3 experiments: Rise, Plateau and Fall. The rats were housed in pairs and exposed to a 12-h/12-h light–dark cycle. Because the task was based on food reinforcement, rats were maintained at 90% of their free-feeding weight. Water was available ad libitum. Food was provided after each familiarization session. Importantly for the present research, previous studies show that rats are able to discriminate speech sounds and language-specific prosodic patterns^56^.

### Stimuli

We used the same stimuli of^13^ (Experiment 1 and 2) which were CCVC (consonant–consonant–vowel–consonant) syllables. See Fig. 1B for the lists of syllables used at familiarization and test. As described in the original article, syllables were recorded by a female native speaker of Russian, which allows all the syllable types used as stimuli. Across experiments, syllables did not differ statistically along different acoustical components, including duration and average pitch. At familiarization, we used 24 syllables, 8 for each experiment. Syllables were classified based on the different degree to which they conformed to the SSP. *Rise* syllables are defined by a prototypical rise of intensity from onset to nucleus, that culminates in the vowel. *Plateau* syllables show a flat intensity from onset to nucleus. Both *Rise* and *Plateau* syllables obey the SSP. *Fall* syllables present a drop of intensity from onset to nucleus, violating the SSP (Fig. 1A). For each experiment, at test, we used 8 syllables: 4 familiar (taken from the list of syllables used at familiarization), 4 novel. Novel test syllables were taken from a familiarization list that was unfamiliar for the animals. At test, in the Rise experiment, rats were presented with *Rise* (familiar) vs. *Fall* (unfamiliar) syllables; in the Fall experiment, rats were presented with *Fall* (familiar) vs. *Rise* (unfamiliar) syllables; in the Plateau experiment, rats were presented with *Plateau* (familiar) vs. *Rise* (unfamiliar) syllables.

### Procedure

We used 8 Letica L830-C response boxes (Panlab S. L., Barcelona, Spain), each one equipped with an infrared detector placed in a pellet feeder that registered nose-poking responses. Stimuli were presented at 84 dB with a loudspeaker (Electro-Voice S-40; response range: 85 Hz–20 kHz) located just outside each response box, behind the feeder, connected to a stereo amplifier (Pioneer A-445). A custom-made software (RatBoxCBC) was used to present stimuli, record nose-poking responses, and deliver food (45 mg-sucrose food pellet).

The animals were not exposed to speech before the beginning of the experiment. The caregiver and the experimenter entered the room (in which rats were housed and tested) one at a time, and were instructed not to emit speech sounds when inside the room. Music and phones were not allowed in the animal facilities.

Prior to the beginning of the experiment, rats underwent a 10-days training phase to learn the nose-poking response. During this phase, each animal was placed individually in the response box for 10 min a day. A sugar pellet was presented every minute, or when the animal poked the feeder with its nose. The experiment consisted of a familiarization phase followed by 4 test sessions. Familiarization and test sessions were structurally identical across experiments, except for the stimuli used.

Familiarization lasted 20 days, and was structured in 1 session of 12-min duration per day. Familiarization sessions were conducted Monday through Friday across 4 weeks. Before stimuli presentation, rats needed a 2-min acquainting period inside the response boxes. During each session, rats were placed individually in the response boxes, and presented with 48 repetitions of the familiarization stimuli (*Rise*, *Plateau* or *Fall* syllables, depending on the experiment). Stimuli were played with a 10-s inter-stimulus interval (ISI). The ISI was kept constant to allow comparisons between the number of nose-poking responses for familiar and unfamiliar stimuli. Rats were not constrained to produce only 1 response after each stimulus, but could respond several times during the ISI. Food reward was delivered during ISI if nose-poking responses were produced by the animals. After familiarization was completed, 4 test sessions took place (in non-consecutive days). Test sessions were structurally similar to familiarization sessions: in each test session, rats were presented with 48 repetitions of the stimuli with a 10-s ISI; test stimuli were 8 syllables, 4 familiar and 4 novel, repeated twice (16 test trials overall). During test sessions, stimuli were presented in a random order with the following restrictions: test items were never presented consecutively, they were interleaved with familiarization items (to avoid response extinction), and no more than 3 familiar items appeared in a row. Food was delivered after nose-poking responses to familiarization syllables only, no food was delivered after nose-poking responses to test syllables.

### Statistical analysis

This section includes additional information about the analyses described in the main text, which were conducted in R (version 4.2.2).

#### Test phase

We conducted two different analyses. To compare discrimination between familiar and novel test items in each experiment, we analyzed sums of nose-poking responses of the 4 test sessions for each rat for each test item. We computed the mean number of responses across rats for each test item, and compared means using paired-samples t-tests.

To compare rats’ overall performance at test across experiments, we analyzed the total number of responses emitted in each experiment. We computed the mean number of responses regardless of the type of test item (responses for familiar and novel items were collapsed) across rats. We compared means across experiments using a one-way ANOVA with experiment (Rise, Plateau, Fall) as between-subject factor, with the *aov* function. Pair-wise t-tests were subsequently computed with Bonferroni correction.

#### Familiarization phase

We compared rats’ responses during the last 5 sessions of familiarization, across experiments. Data were number of responses of each familiarization session for each rat. We computed the mean number of responses across rats and across familiarization sessions. We compared means across experiments using a one-way ANOVA with familiarization group (Rise, Plateau, Fall) as between-subject factor, with the *aov* function. Pair-wise t-tests were subsequently computed with Bonferroni correction. We conducted this same analysis to compare rats’ responses during the first 5 sessions of familiarization, across experiments.

## Data Availability

Data, scripts for analysis, stimuli, stimuli duration, and schematic representation of the experimental setting are available at Open Science Framework: https://osf.io/7hjzv/.
